# Supplementary material for: TGF-β Isoforms and Local Environments Greatly Modulate Biological Nature of Human Retinal Pigment Epithelium Cells
Source: Bioengineering (Basel). 2024 Jun 7;11(6):581. doi: 10.3390/bioengineering11060581 (PMC11201039; doi:10.3390/bioengineering11060581)
Supplement: Supplementary file 1 [file bioengineering-11-00581-s001.zip › bioengineering-2998830-supplementary.pdf]

Supplementary File
Table S1

ZO-1	Taqman	GCCACTACAGTATGACCATCC	GCTGGCTTATTCTGAGATGGA	/56-FAM/ACTGAATTA/ZEN/CCTTCACCATGTGCTCCC/3IABkFQ/	NM_175610	4-25
αSMA	Taqman	CTGTTGTAGGTGGTTTCATGGA	AGAGTTACGAGTTGCCTGATG	/56-FAM/AGACCCTGT/ZEN/TCCAGCCATCCTTC/3IABkFQ/	NM_001613	8-9
Col1	Taqman	TTCTGTACGCAGGTGATTGG	GACATGTTCAGCTTTGTGGAC	/56-FAM/TCGAGGGCC/ZEN/AAGACGAAGACATC/3IABkFQ/	NM_000088	1-2a
HIF1α	Taqman	CCGTCATCTGTTAGCACCAT	GCTCACCATCAGTTATTTACGTG	/56-FAM/TCTAGACCA/ZEN/CCGGCATCCAGAAGT/3IABkFQ/	NM_010431	2-3
PGC1α	Taqman	GAGTCTGTTATGGAGTGACATCG	TGTCTGTATCCAAGTCGTTCAC	/56-FAM/ACCAGCCTC/ZEN/TTTGCCCAGATCTTC/3IABkFQ/	NM_013261	1-2
